# Paralytic Rabies Encephalomeningomyelitis: A Tale of Two Cases With Emphasis on Imaging Findings and Relevant Literature Review

**DOI:** 10.7759/cureus.113057

**Published:** 2026-07-20

**Authors:** Ketki Kedar, Ami V Patel, Chetana Ratnaparkhi, Avinash Dhok, Sunita Kumbhalkar

**Affiliations:** 1 Radiodiagnosis, All India Institute of Medical Sciences, Nagpur, Nagpur, IND; 2 Radiology, All India Institute of Medical Sciences, Nagpur, Nagpur, IND; 3 Radiodiagnosis and Interventional Radiology, All India Institute of Medical Sciences, Nagpur, Nagpur, IND; 4 General Medicine Cardiology, All India Institute of Medical Sciences, Nagpur, Nagpur, IND

**Keywords:** acute disseminated encephalomyelitis, case report, magnetic resonance imaging, paralytic rabies, post-exposure prophylaxis, rabies encephalomeningomyelitis

## Abstract

Imaging documentation of rabies encephalitis remains rare because of the progressive and fatal course of the disease. We present two cases of paralytic rabies encephalomeningomyelitis, showing varied imaging findings, one in a two-year-old girl whose magnetic resonance imaging (MRI) illustrated typical features, and another case of a 65-year-old female with atypical MRI findings. These two cases emphasize that classical rabies shows predominant grey-matter involvement, whereas an atypical presentation, particularly in elderly patients with underlying comorbidities, shows a predilection for white matter, in contrast to the typical form. The identification of this varied imaging spectrum is crucial for prompt diagnosis, especially in endemic zones. The rarity of antemortem neuroimaging in rabies, with detailed documentation of typical and atypical cases, enriches the limited diagnostic literature and improves early recognition of the disease’s imaging spectrum.

## Introduction

Rabies is a nearly fatal viral encephalitis caused by a neurotropic RNA virus of the genus Lyssavirus, transmitted by bite of an infected canine through saliva [1]. Rabies continues to be a major public health concern, resulting in nearly 59,000 deaths annually worldwide [1]. India accounts for almost one-third of these deaths. Children are especially vulnerable, as bites may go unnoticed or reported late [1]. Clinically, rabies manifests as a common encephalitic form, characterized by hydrophobia, aerophobia, and autonomic instability, and a less common paralytic form presenting with ascending flaccid paralysis that mimics Guillain-Barré syndrome (GBS) or acute disseminated encephalomyelitis (ADEM) [2,3]. Owing to its rapidly progressive course and almost invariably fatal outcome, magnetic resonance imaging (MRI) in rabies is infrequently reported. We present two cases of rabies with typical and atypical MRI manifestations, emphasizing their diagnostic value and adding to the limited literature on rabies survivors.

## Case presentation

Case 1

A two-year-old girl presented with high-grade fever, altered sensorium, and nystagmus for four days. She had a Grade III dog bite on the face 15 days prior. Post-exposure prophylaxis (PEP) was given as rabies vaccine (0, 3, 7, and 14 doses); however, the 28th dose and rabies immunoglobulin (RIG) were not administered. There was no hydrophobia, aerophobia, or dysphagia. On admission, her Glasgow Coma Scale score was 9/15. Neurological examination revealed generalized hypotonia, muscle power of 3/5 in all four limbs, absent deep tendon reflexes, and bilateral extensor plantar responses. The laboratory parameters are mentioned in Table 1.

**Table 1:** Laboratory investigations. WBC = white blood cell count; APTT = activated partial thromboplastin time; ESR = erythrocyte sedimentation rate; CRP = C-reactive protein; CSF = cerebrospinal fluid

Investigation	Result	Reference range
WBC	22.18 × 10³/μL (raised)	4–10
Hemoglobin	9.1 g/dl (reduced)	12–15
Platelet count	535 × 10³/μL	150–450
APTT	28.6 seconds	28.04–37.83
ESR	27 (raised)	-
Serum sodium	129 mmol/L	135–145
Serum calcium, ionic	1.1 mmol/L	1.13–1.31
Serum calcium, total	9.3 mg/dL	8.4–10.2
CRP, quantitative	8.9 mg/L(raised)	<5.0
CSF routine microscopy	Colourless, clear, no coagulum, total count nil	-
CSF Gram stain	Occasional pus cell, no organism seen	-
CSF sugar	68.2 mg/dL	60–80
CSF total protein	34.4 mg/dL	15–45

**Figure 1:**
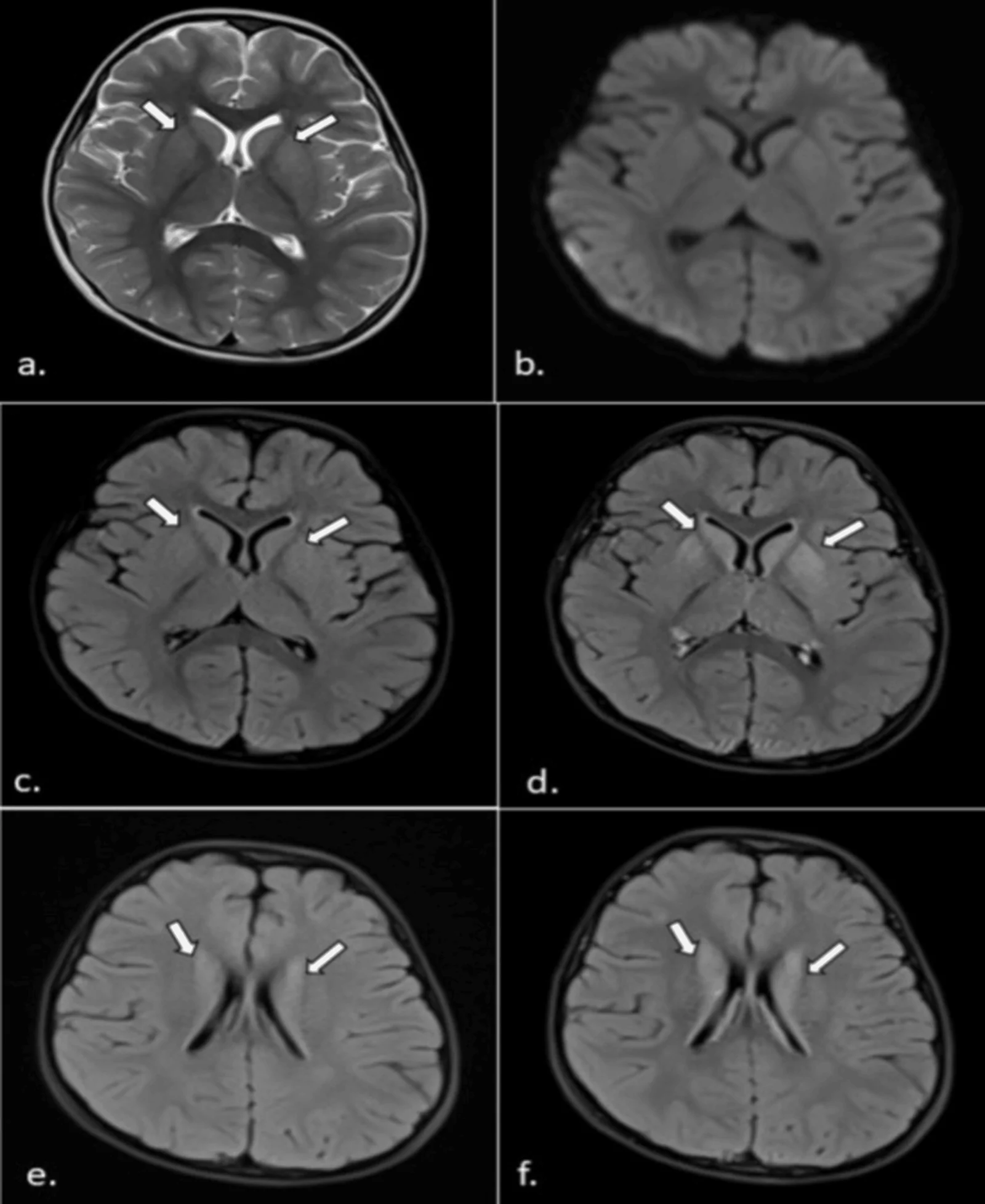
Magnetic resonance imaging of the brain. Axial T2-weighted (a), diffusion-weighted imaging (b), pre-contrast fluid-attenuated inversion recovery (FLAIR) (c), and post-contrast FLAIR (d) image at the level of the basal ganglia showing hyperintensity involving the heads of the bilateral caudate nuclei and bilateral lentiform nuclei (a, c) with no diffusion restriction (b) and showing post-contrast enhancement (d) (arrows). Pre-contrast (e) and post-contrast (f) axial FLAIR image at the level of the caudate nucleus showing hyperintensity involving the bilateral caudate nuclei with enhancement (arrows).

**Figure 2:**
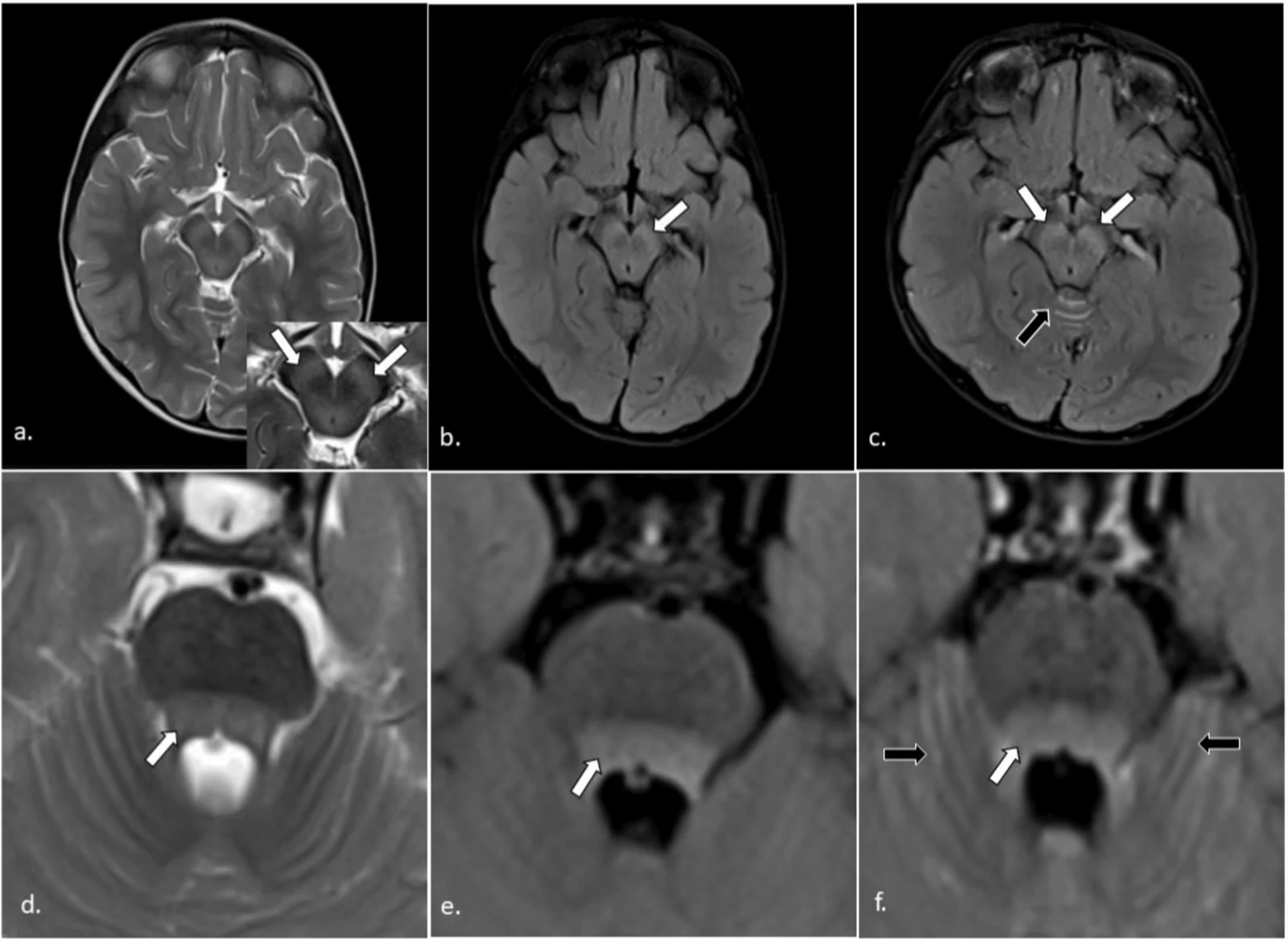
Magnetic resonance imaging of the brain. Axial T2-weighted (T2W) (a), pre-contrast fluid-attenuated inversion recovery (FLAIR) (b), and post-contrast FLAIR (c) images at the level of the midbrain showing hyperintensity involving the bilateral cerebral peduncles with enhancement (arrows) and leptomeningeal enhancement along the vermis (black arrow). Axial T2W (d), pre-contrast FLAIR (e), and post-contrast FLAIR (f) at the level of the pons showing hyperintensity involving the dorsal pons with enhancement( arrows) along the cerebellar folia (black arrows).

Rabies polymerase chain reaction (PCR) testing of cerebrospinal fluid (CSF), saliva, and serum was negative; however, CSF antibody titers were elevated (1:16). MRI of the brain showed nearly symmetrical bilateral T2/fluid-attenuated inversion recovery (FLAIR) hyperintensities involving the caudate nuclei, lentiform nuclei, cerebral peduncles, and dorsal pons, with meningeal enhancement along the cerebellar folia. However, these areas showed no diffusion restriction (Figures 1, 2).

**Figure 3:**
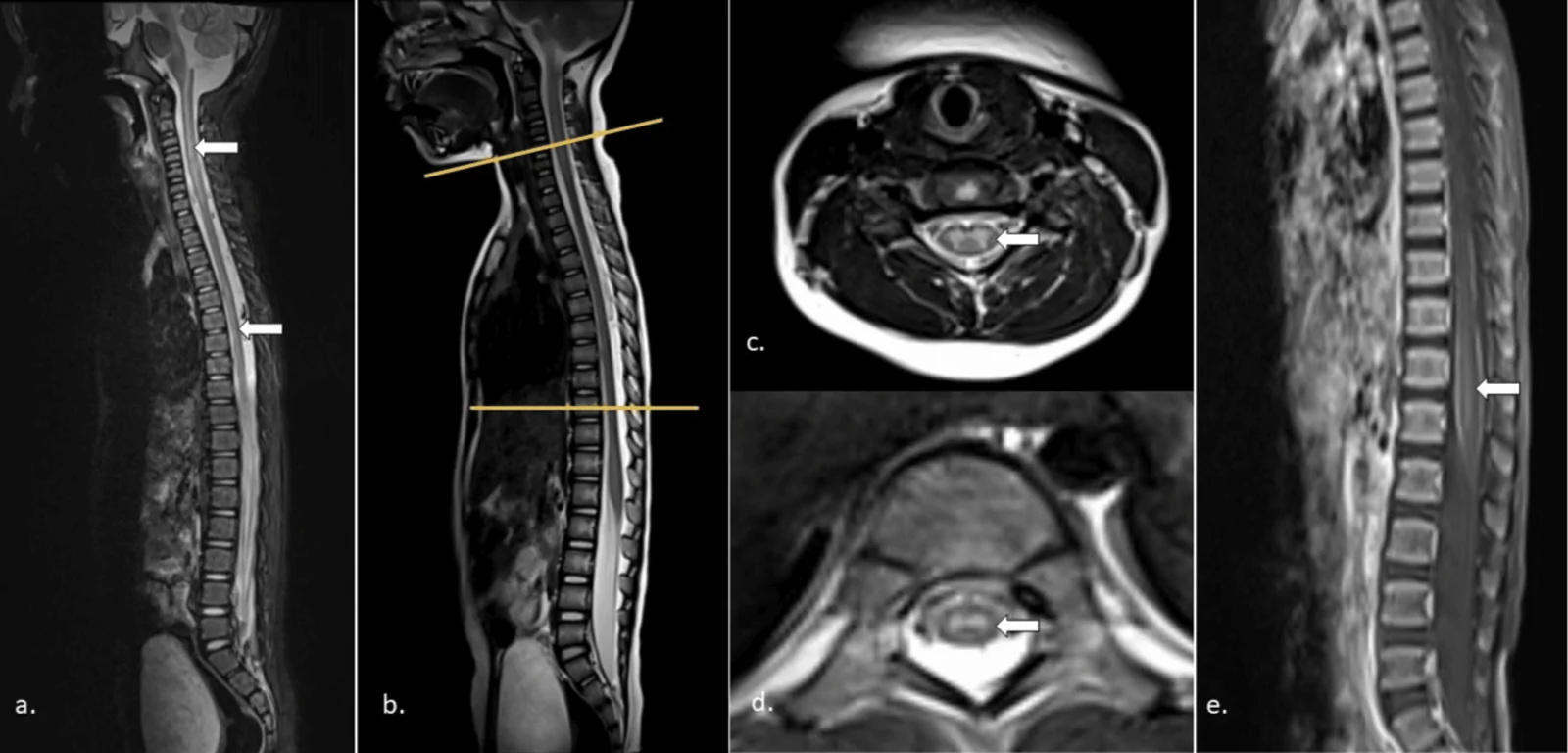
Magnetic resonance imaging of the spine. (a) Whole-spine short-TI inversion recovery (STIR) sagittal image showing STIR hyperintensity in the cervicodorsal spinal cord (arrows). (b) Whole-spine T2-weighted sagittal image showing subtle T2 hyperintensity in the cervicodorsal spinal cord. (c and d) Axial T2-weighted images at the cervical and dorsal spinal cord, respectively, showing central grey-matter hyperintensity (arrows). (e) Post-contrast sagittal dorsolumbar cord image showing post-contrast enhancement involving the conus (arrow).

MRI of the spine showed a long-segment, non-enhancing, intramedullary, hyperintense signal seen in the cervico-dorsal spinal cord, extending from C2 and reaching up to C7 vertebral levels with leptomeningeal enhancement extending from dorsal vertebral D3 level to the conus medullaris (Figure 3).

In view of history and MRI findings, the diagnosis of rabies encephalomeningomyelitis was made. The child was treated with intravenous immunoglobulin at a dose of 2 g/kg along with acyclovir. The patient responded to the treatment well with a favorable neurological recovery at the two-year follow-up, making her a rare survivor of rabies encephalitis.

Case 2

A 65-year-old woman presented with rapidly progressive lower motor neuron sensorimotor quadriparesis, requiring mechanical ventilation. Family members reported a recent dog bite on the right upper limb. She had received a full course of rabies vaccination and RIG. On examination, flaccid quadriparesis, hyporeflexia, and reduced power in all four limbs were identified. The laboratory parameters are mentioned in Table 2.

**Table 2:** Laboratory investigations. WBC = white blood cell count; CRP = C-reactive protein; CSF = cerebrospinal fluid

Investigation	Result	Reference range
WBC	14.97 ×10³/μL (raised)	4–10
Neutrophils	88.7% (raised)	40–80
Lymphocyte	10.1%	20–40
Monocytes	1.1%	2–10
Eosinophils	0.0%	1–6
Basophil	0.1%	0–1
Hemoglobin	8.8 g/dL (reduced)	12–15
Platelet count	316 × 10³/μL	150–450
Serum sodium	129 mmol/L (reduced)	135–145
Total serum protein	5.65 g/dL	6.6–8.7
Serum albumin	2.28 g/dL (reduced)	3.5–5.0
CRP (quantitative)	235 mg/L (markedly raised)	<5
CSF routine microscopy	Colorless, clear, no coagulum, total count Nil	-
CSF cell count	110 cells/mm³, 70% lymphocytes	
CSF Gram stain	Occasional pus cell, no organism seen	-
CSF sugar	68.2 mg/dL	40–70
CSF total protein	89.8 mg/dL (raised)	15–45

**Figure 4:**
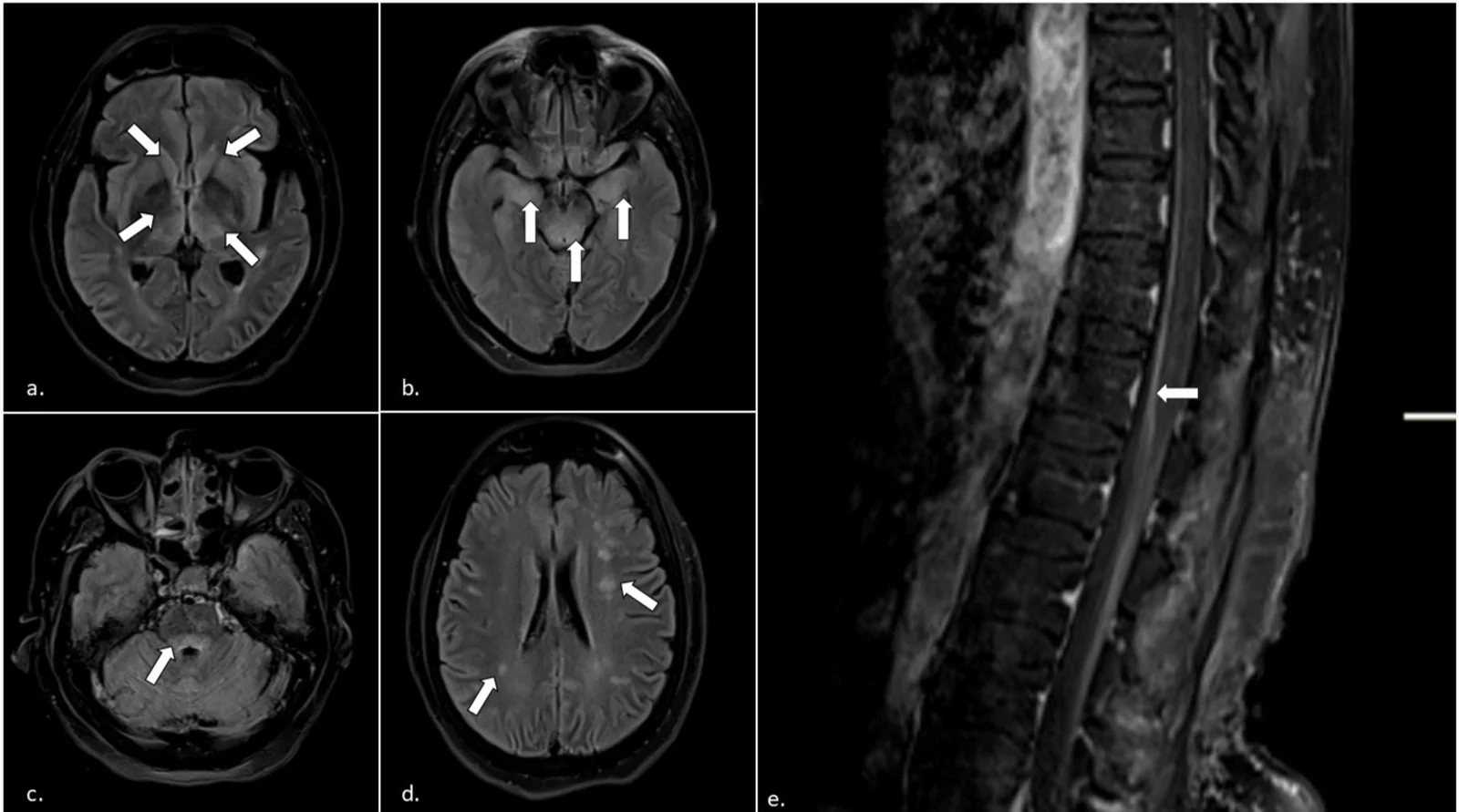
Magnetic resonance imaging of the brain and spine. (a) Axial fluid-attenuated inversion recovery (FLAIR) image at the level of the basal ganglia showing hyperintensity involving the heads of the bilateral caudate nuclei, thalami and putamina (arrows). (b) Axial FLAIR image at the level of the midbrain showing hyperintensity involving the dorsal aspect of the midbrain and bilateral hippocampi (arrows). (c) Axial FLAIR image at the level of the pons showing hyperintensity involving the dorsal pons (arrows). (d) Axial FLAIR image showing bilateral asymmetrical white-matter FLAIR hyperintensities involving the bilateral corona radiata and periventricular white matter (arrows). (e) Post-contrast sagittal image at the level of the conus showing enhancement along the anterior aspect of the conus (arrows).

CSF analysis showed lymphocytic pleocytosis and positive rabies antibody titers from CSF. MRI of the brain demonstrated symmetrical, non-enhancing T2/FLAIR hyperintensities involving the bilateral lentiform nuclei, thalami, hippocampi and parahippocampal gyri, and dorsal brainstem. Atypical non-enhancing white matter lesions were present in the bilateral frontal, temporal, occipital, and left parietal lobes. MRI of the spine showed focal enhancement along the anterior aspect of the conus (Figure 4).

Based on the clinical history, serological and imaging findings, a diagnosis of rabies encephalomeningomyelitis was made. The patient was kept on empirical treatment consisting of intravenous (IV) fluids, IV antibiotics, IV antivirals, and IV anticonvulsants; however, the patient’s neurological condition deteriorated and succumbed after two weeks of hospitalization.

## Discussion

Human rabies is endemic in India. Approximately 18,000-20,000 deaths are reported annually, particularly from rural sectors due to limited access to post-exposure care [1]. Facial bites carry a higher risk of treatment failure because of proximity to the central nervous system (CNS) and a shorter incubation period, as seen in our pediatric case [1].

The PEP for Grade II and III bites, comprising wound washing, complete dose-weighted adjusted vaccination, and RIG, has been recommended [4]. The missed day 28 vaccine dose in the first case might have led to an incomplete humoral response and thereby CNS spread.

The pathological hallmark of rabies encephalitis is neuronal involvement with relative sparing of axons and white matter, manifesting on MRI as T2/FLAIR hyperintensity and post-contrast enhancement in grey matter structures, including the basal ganglia (particularly the caudate and putamen), thalami, brainstem, hippocampi, and hypothalamus [5]. Meningeal enhancement and spinal cord involvement, particularly in paralytic rabies, are additional features [3]. It is reported that rabies initially affects the brainstem, followed by the basal ganglia and thalamus [5]. Another characteristic imaging feature is the typically absent diffusion restriction, which helps distinguish rabies from acute infarction and herpes simplex encephalitis (HSE) [6,7].

White matter involvement is unusual in rabies and may complicate diagnosis, as classic rabies encephalitis typically lacks it [1,2]. However, cases involving the white matter and centrum semiovale have been reported, as in the second case, suggesting an atypical manifestation [5]. Alternatively, age-related small-vessel disease or coexisting ADEM post-vaccination may explain these findings.

ADEM typically follows viral infection or vaccination and, on imaging, demonstrates asymmetric multifocal white matter lesions involving subcortical U-fibers [2]. Although deep grey matter involvement may occur, bilateral symmetrical grey matter abnormalities are uncommon. In the first case, the absence of white matter lesions together with symmetrical grey matter and brainstem involvement strongly favored rabies over ADEM [2]. GBS is another differential for paralytic rabies; however, GBS primarily affects the peripheral nervous system without brain parenchymal abnormalities [8].

Important imaging differentials also included Japanese encephalitis, HSE, metabolic encephalopathies, and stroke [5,7]. Because HSE is treatable and may mimic rabies, empirical acyclovir remains justified until rabies is confirmed [7]. Antemortem MRI-documented rabies remains rare, with most reports originating from India and the rest from Southeast Asia [5]. Survivors are exceptionally rare; the first patient further adds to this sparse literature [9].

## Conclusions

These cases underscore the importance of imaging in rabies encephalitis. The presence of classical bilateral, symmetrical grey matter signal changes in the basal ganglia and brainstem on MRI should prompt diagnosis of paralytic rabies; however, atypical presentation as seen in the second case should also raise suspicion in patients with a dog bite, particularly in endemic areas. Given the rarity of antemortem neuroimaging data in rabies, detailed reporting of such cases continues to add to the scarce diagnostic literature meaningfully.
